# A retrospective observational study of ibrutinib in chronic lymphocytic leukaemia in a real-life setting in France using the national claims database (OSIRIS)

**DOI:** 10.1007/s00277-024-05859-w

**Published:** 2024-07-05

**Authors:** Sylvain Choquet, Clarisse Marchal, Floriane Deygas, Marine Deslandes, Nahid Macher, Gérard de Pouvourville, Vincent Levy

**Affiliations:** 1 Hôpital Pitié salpêtrière AP-HP, Paris, France; 2 PELYON, Lyon, France; 3 Janssen-Cilag France, Issy-les-Moulineaux, France; 4 ESSEC, Cergy-Pontoise, France; 5 Hôpital Avicenne AP-HP, Bobigny, France

**Keywords:** Chronic lymphocytic leukaemia, Claims database, Ibrutinib, Treatment pattern

## Abstract

**Background:**

Ibrutinib is a Bruton’s tyrosine kinase inhibitor indicated for the first-line treatment and relapse of chronic lymphocytic leukaemia (CLL), Waldenström’s macroglobulinemia (WM) and mantle cell lymphoma (MCL). This study aimed to describe the characteristics of CLL patients treated with ibrutinib and its effectiveness, safety, and treatment pattern in real life.

**Methods:**

All patients covered by the general health scheme (approximately 80% of the French population) with a first ibrutinib dispensation from August 1, 2017 (date of reimbursement in France) to December 31, 2020, were identified in the French National Health Insurance database (SNDS). An algorithm was developed to identify the disease (CLL, MCL or WM) for which ibrutinib was prescribed. This article focused on CLL patients. The time to next treatment (TTNT) was plotted using Kaplan‒Meier curves.

**Results:**

During this period, 6,083 patients initiated ibrutinib, among whom 2,771 (45.6%) patients had CLL (mean age of 74 years; 61% of men). At ibrutinib initiation, 46.6% of patients had a cardiovascular comorbidity. Most patients (91.7%) were not hospitalized during the exposure period for one of the cardiovascular or bleeding events studied. Hospitalizations were more frequent in patients with a cardiovascular comorbidity (5.9% versus 11.0%, p-value < 0.0001) and aged over 70 (5.9% versus 9.4%, p-value < 0.0001). The median TTNT was not reached.

**Conclusion:**

This is one of the largest cohorts of ibrutinib-treated patients in the world. The profile of CLL patients treated with ibrutinib was in accordance with the marketing authorization and reimbursement. This study confirmed effectiveness and safety data.

**Supplementary Information:**

The online version contains supplementary material available at 10.1007/s00277-024-05859-w.

## Introduction

Chronic lymphocytic leukaemia (CLL) is an indolent non-Hodgkin lymphoma and the most common type of leukaemia in adults (between 25% and 30% of leukaemia cases) [1–3]. Each year in Europe, approximately 5 per 100,000 persons are diagnosed with CLL [4]. In France in 2018, the number of new cases of CLL was estimated at 4,674, including 59% men [5]. Indeed, men are known to be at higher risk than women, with a world standardized incidence ratio (SIR) of 4.0 and 2.1 per 100,000 person-years, respectively (male/female ratio, 1.9) [5, 6]. Life expectancy for patients with CLL varies from 6.5 to 10 years or more, depending on the stage at which the disease is diagnosed.

Numerous frontline therapies are available for treating CLL, including immune-chemotherapies and targeted therapies, such as Bruton tyrosine kinase inhibitors (BTKis), phosphoinositide 3-kinase inhibitors (PI3Kis) and B-cell lymphoma 2 inhibitors (BCL2is). Ibrutinib is a first-in-class BTKi that has been shown to have single-agent activity against many B-cell malignancies, including CLL, mantle cell lymphoma (MCL) and Waldenström’s macroglobulinemia (WM) [7–9]. Several targeted therapies have been approved for the treatment of adult patients with CLL in frontline and/or relapse/refractory settings, including ibrutinib, idelalisib, venetoclax, acalabrutinib and zanubrutinib.

From February 1st, 2014, to July 31st, 2017, ibrutinib was available through temporary use authorization (TUA), a French program allowing early access to medicines. Since August 1st, 2017, ibrutinib has been available in community pharmacies in France and reimbursed for the following indications [10, 11]: treatment of adult patients with CLL who have received at least one prior therapy (L2+); first-line treatment of adult patients with CLL (L1) in the presence of 17p deletion or TP53 mutation; treatment of adult patients with relapsed or refractory MCL (L2+); and treatment of adult patients with WM who have received at least one prior therapy (L2+).

Since November 17th, 2020, the indication has been extended to include the first-line treatment of patients with CLL who are ineligible for full-dose fludarabine treatment and do not present with 17p deletion or TP53 mutation.

In CLL, initial studies of the single agent ibrutinib have reported that it is well tolerated [12, 13]. However, an association with some cardiovascular events has been reported, such as hypertension or arrythmia [14–16]. These cardiovascular events have also been reported for other BTKis, such as acalabrutinib and zanubrutinib, during clinical trials [17, 18].

The OSIRIS study aimed to describe the characteristics of CLL patients treated with ibrutinib, to determine the treatment pattern and safety of ibrutinib and to estimate the effectiveness of ibrutinib in a real-life setting using claims data from the French National Health Insurance System database (Système National des Données de Santé; [SNDS]). The impact of a cardiovascular comorbidity on effectiveness was also explored.

This article focused on patients with CLL, constituting the largest group of ibrutinib users in France.

## Method

### Data source

This retrospective observational cohort study was conducted using data from the French health insurance database (SNDS). Data recorded include anonymous individual information on patients’ sociodemographic characteristics (gender, month and year of birth, month and year of death if applicable), nonhospital reimbursed healthcare expenditures with date and code (visits and medical procedures, laboratory tests, drugs, and medical devices, but not the corresponding medical indication or results) and all hospital discharge summaries (ICD-10 diagnoses codes for all medical, obstetric, and surgery hospitalizations with the date and duration of hospitalization, medical procedures, hospital department, and cost coding system) [19].

### Study population and study design

The OSIRIS study involved all patients with at least one ibrutinib dispensation from August 1st, 2017 (date of ibrutinib availability in community pharmacy) to December 31st, 2020 (inclusion period). The date of first ibrutinib dispensation was defined as the index date, and the period between January 1st, 2006 (start date of SNDS data history) and the index date was defined as the preindex period. Patients were followed-up until December 31st, 2020, death or the last patient’s health record (i.e., last care recorded in the database before a period of 6 months without any reimbursed care), whichever occurred first.

Patients not covered by the general health insurance scheme and local mutualist section (approximately 20% of the French population) during both the preindex and inclusion periods (i.e., from 2006 to 2020) were excluded (data from other health insurance schemes not available throughout the study period). Patients without any other care recorded in the SNDS after their first dispensation of ibrutinib (except in case of death) were also excluded. Then, an algorithm was developed to identify the disease for which ibrutinib was used (CLL, MCL or WM), using ICD-10 codes from the long-term disease (LTD) status and main, related, and associated hospital diagnoses. The disease was identified based on the last recorded diagnosis of CLL, MCL or WM (ICD codes C911, C831 and C880, respectively) in the preindex period or, if no diagnosis was recorded before the index date, on the first recorded diagnosis up to 30 days after the last dispensation of ibrutinib.

For this article, only patients with a CLL diagnosis who initiated ibrutinib from August 1st, 2017, were included in the study population.

### Variables

The pattern of ibrutinib use was studied using algorithms defining the line of treatment (first or second or more line) and the ibrutinib regimen (Online Resource 1 and 2).

#### Primary endpoint

The time to next treatment (TTNT), defined as the time between the index date and the first dispensation of the next treatment or death, was used to assess ibrutinib effectiveness as a proxy for progression-free survival (PFS).

### Secondary endpoints

The time to discontinuation (TTD), defined as the time between the index date and permanent discontinuation or death, was also studied. The time-free interval (TFI) was defined as the time in days between the end of the last regimen of ibrutinib and the first dispensation of the next treatment.

Diagnoses recorded in LTD status and hospital discharges in the 12 months before ibrutinib initiation were used to identify the comorbidities and compute the Charlson comorbidity index [20]. To identify cardiovascular comorbidities, markers other than diagnoses were also considered, such as the use of pacemakers, antiplatelet drugs and/or anticoagulant agents (probabilistic markers).

Hospitalizations (main and related discharge diagnoses) for the following cardiovascular or bleeding events occurring during the follow-up period were described: atrial fibrillation, hypertension, bleeding, cardiac arrest, paroxysmal tachycardia, and heart failure. The time between ibrutinib initiation and the first hospitalization for one of these events was estimated.

### Statistical analyses

To describe all the concomitant treatments received over the first regimen, these variables were described only for patients with a complete first ibrutinib regimen. Treatment history was described in the same population, and the next treatments were only described for those with permanent discontinuation of ibrutinib. All other analyses were performed in the overall study population.

Kaplan‒Meier curves were used to plot the TTNT and TTD, overall and according to the presence of a cardiovascular comorbidity at the index date. Patients were censored at the end of the study period or upon loss to follow-up. A Cox model was applied to identify the explanatory factors for the TTNT.

All statistical analyses were performed using SAS (SAS Institute, North Carolina, US), version 9.4.

## Results

### Selection of the population

**Fig. 1:**
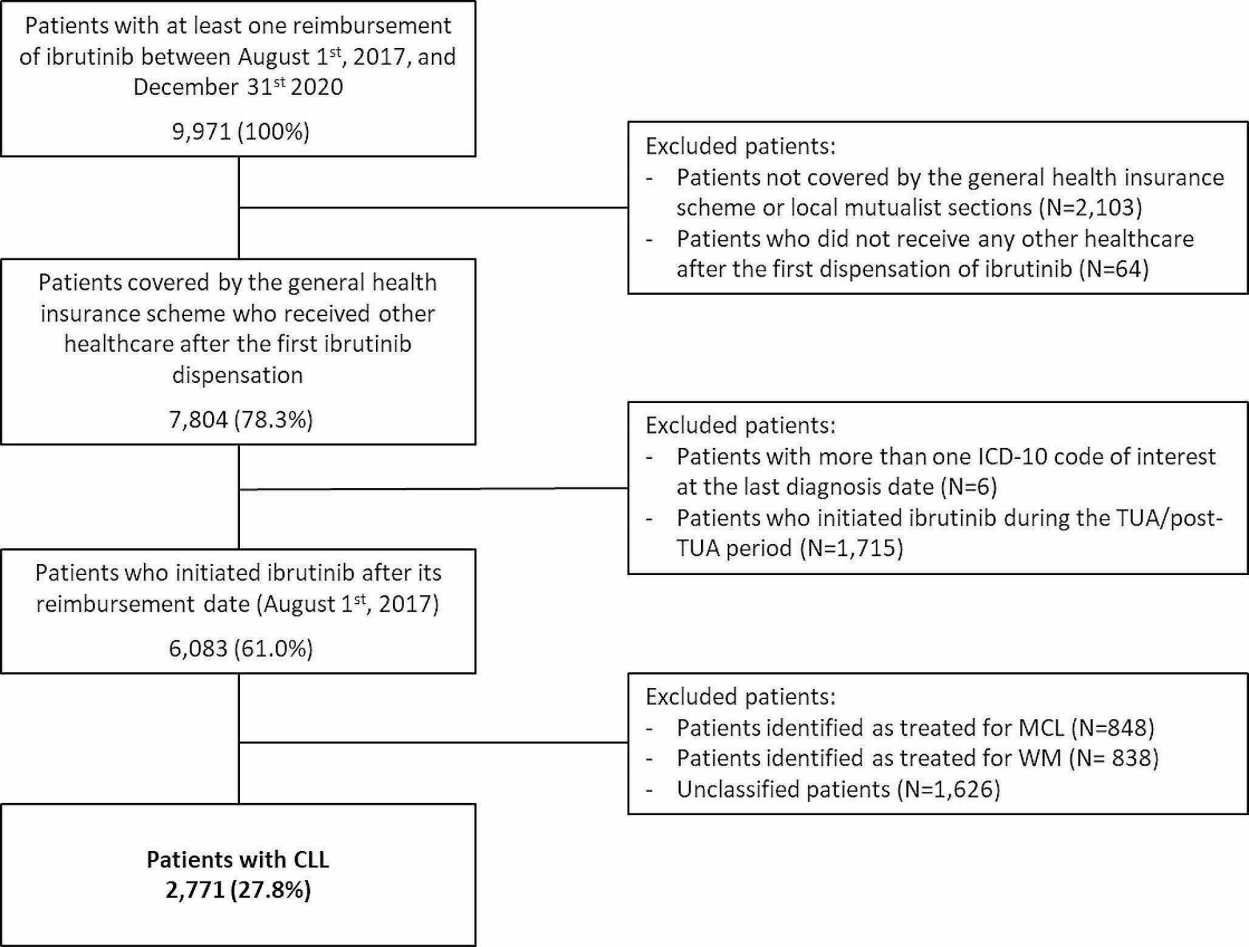
Study flowchart

The selection criteria led to the identification of 6,083 (61.0%) patients who had initiated ibrutinib during the inclusion period, of whom 2,771 (45.6%) had CLL (Fig. 1). The results presented hereafter relate to this cohort of 2,771 CLL patients who received their first dispensation of ibrutinib from August 1st, 2017.

### Characteristics of the cohort (*N* = 2,771)

Patients were followed for a median of 16.2 (Q1-Q3: 7.0–27.0) months. Most patients were male (*n* = 1,695, 61.2%) (Table 1). The median age at ibrutinib initiation was 74 years (Q1–Q3: 67.0–80.0).

At ibrutinib initiation, most patients (*n* = 1,953, 70.5%) had a high comorbidity burden, with a Charlson comorbidity index ≥ 5. The most frequent comorbidity was cardiovascular disease (*n* = 1,291, 46.6%). Most of these patients (*n* = 1,008, 78.1%) had a marker of cardiovascular disease other than a diagnosis code (i.e., treatment or pacemaker). A solid tumour diagnosis was identified in 19.0% of patients (*n* = 527), and the three most frequent diagnoses were skin (*n* = 139, 26.4%), male genital organ (*n* = 102, 19.4%) and breast (*n* = 69, 13.1%) cancer.

**Table 1:** Demographic and clinical characteristics of patients with CLL at ibrutinib initiation

	CLL (*N* = 2,771)
**Duration of follow-up (months)**	
Mean (SD)	17.6 (11.7)
Median (Q1-Q3)	16.2 (7.0–27.0)
Min–Max	0.1–41.1
**Reason for end of follow-up**	
End of study	2,211 (79.8%)
Death	530 (19.1%)
Lost to follow-up	30 (1.1%)
**Sex**	
Male	1,695 (61.2%)
**Age (in years)**	
< 40	9 (0.3%)
40–49	43 (1.6%)
50–59	227 (8.2%)
60–69	650 (23.5%)
70–79	1,072 (38.7%)
80–89	703 (25.4%)
≥ 90	67 (2.4%)
**Age (in years)**	
Mean (SD)	72.9 (10.1)
Median (Q1 - Q3)	74.0 (67.0–80.0)
**Death during follow-up**	530 (19.1%)
**Place of death**	
Out of hospital	113 (21.3%)
In hospital	417 (78.7%)
**Number of comorbidities**	
0	1,175 (42.4%)
1	1,013 (36.6%)
2	451 (16.3%)
3	113 (4.1%)
4	19 (0.7%)
**Comorbidities**	
Chronic disease of the liver and cirrhosis	44 (1.6%)
Cardiovascular disease	1,291 (46.6%)
*Disabling stroke*	65 (2.3%)
*Chronic arterial disease*	113 (4.1%)
*Heart failure, arrhythmia, valvular and severe congenital heart disease*	368 (13.3%)
*Severe hypertension*	465 (16.8%)
*Coronary artery disease*	275 (9.9%)
*Markers of cardiovascular disease*	1,008 (36.4%)
Severe chronic respiratory failure	276 (10.0%)
Severe chronic kidney disease	192 (6.9%)
Solid tumour	527 (19.0%)
**Charlson comorbidity index**	
0	5 (0.2%)
1–2	65 (2.3%)
3–4	748 (27.0%)
≥ 5	1,953 (70.5%)

### Pattern of ibrutinib use in CLL (*n* = 2,771)

Most patients had initiated ibrutinib as L2+ (*n* = 2,221, 80.2%) (Table 2). Over the study period, the proportion of patients who initiated ibrutinib as L1 increased (from 15.0% in 2017 to 29.1% in 2020). Most patients in L1 (*n* = 449, 81.6%) and L2+ (*n* = 1,816, 81.8%) used ibrutinib as a monotherapy.

**Table 2:** Description of the line of ibrutinib treatment in patients with CLL

	CLL (*N* = 2,771)
**Line of treatment and concomitant therapies, ** ***n*** **(%)**	
L1	550 (19.8%)
Ibrutinib in monotherapy	449 (81.6%)
Ibrutinib + other CLL-specific treatment	92 (16.7%)
Ibrutinib + rituximab	5 (0.9%)
Ibrutinib + obinutuzumab	4 (0.7%)
L2+	2,221 (80.2%)
Ibrutinib in monotherapy	1,816 (81.8%)
Ibrutinib + other CLL-specific treatment	394 (17.7%)
Ibrutinib + bendamustine + rituximab	11 (0.5%)
**Time since first treatment for CLL (in years)**	
N (%)	2,221 (80.2%)
Mean	5.3 (3.5)
Median (Q1 - Q3)	4.8 (2.5–7.9)
Min–Max	0.0-14.7
**Patients who have completed their first ibrutinib regimen**	1,061 (38.3%)
**Number of ibrutinib dispensations during the first ibrutinib regimen completed**	
Mean (SD)	7.5 (7.0)
95% CI	[7.0 ; 7.9]
Median (Q1 - Q3)	5.0 (2.0–11.0)
**Length of the first ibrutinib regimen completed (in days)**	
Mean (SD)	268.5 (249.3)
95% CI	[253.4 ; 283.5]
Median (Q1 - Q3)	181.0 (75.0-391.0)
**Time to cotherapy initiation (in days)**	
N (%)	261 (24.6%)
Mean (SD)	135.7 (216.7)
Median (Q1 - Q3)	27.0 (0.0-179.0)
**Length of discontinuation period between first and second ibrutinib regimen (in days)**	
N (%)	117 (4.2%)
Mean (SD)	121.7 (24.0)
95% CI	[117.3 ; 126.1]
Median (Q1 - Q3)	115.0 (103.0-141.0)

### Pattern of use among patients who had completed a first regimen of ibrutinib (*n* = 1,061)

Less than half (*n* = 1,061; 38.3%) of CLL patients had completed their first regimen of ibrutinib, with a median duration of approximately 6.0 (Q1-Q3: 2.5–12.9) months and a median of 5.0 ibrutinib dispensations (Q1-Q3: 2.0–11.0).

Among these 1,061 patients, most had received cancer treatment before ibrutinib initiation (*n* = 882, 83.1%) (Table 3) and the most frequent last treatment received was rituximab (*n* = 359, 33.8%). Among the 481 patients (45.3%) who permanently discontinued ibrutinib and did not die by the end of the study period, 64.2% (*n* = 309) received another treatment. The most frequent next treatment received was venetoclax (*n* = 101, 21.0%).

**Table 3:** Treatments before and after ibrutinib exposure in patients with CLL who completed their first ibrutinib regimen

	CLL (*N* = 1,061)	
**Previous cancer treatments**		
Chemotherapy and/or immunotherapy		866 (81.6%)
Targeted therapy		68 (6.4%)
Infusion session		719 (67.8%)
Session with bendamustine and/or rituximab and/or obinutuzumab		677 (63.8%)
Session with CLL-specific treatment (except bendamustine, rituximab and obinutuzumab)		37 (3.5%)
Session with intra-DRG^a^ or other treatment		312 (29.4%)
**Last treatment dispensed before index date**		
**Time since previous treatment (in days)**		
N (%)		882 (83.1%)
Mean (SD)		891.9 (868.1)
Median (Q1 - Q3)		685.0 (150.0–1,339.0)
**Chemotherapy and/or immunotherapy extra DRG**		620 (58.4%)
Rituximab		359 (33.8%)
Bendamustine		228 (21.5%)
Fludarabine		93 (8.8%)
Obinutuzumab		25 (2.4%)
Doxorubicin		2 (0.2%)
**Targeted therapy extra-DRG**		2 (0.2%)
Bortezomib		2 (0.2%)
**Session with intra-DRG or other treatment**		128 (12.1%)
**Next cancer treatment**		
**Living patients at the end of the study period with a permanent discontinuation**		481 (45.3%)
**Time-free interval (in months)**		
N (%)		309 (64.2%)
Mean (SD)		3.7 (5.2)
Median (Q1 - Q3)		1.1 (0.4–4.7)
**Chemotherapy and/or immunotherapy extra-DRG**		93 (19.3%)
Rituximab		77 (16.0%)
Obinutuzumab		11 (2.3%)
Bendamustine		10 (2.1%)
Doxorubicin		1 (0.2%)
**Targeted therapy extra-DRG**		1 (0.2%)
Acalabrutinib		1 (0.2%)
**Targeted therapy intra-DRG, available in outpatient pharmacy**		112 (23.3%)
Venetoclax		101 (21.0%)
Idelalisib		10 (2.1%)
Lenalidomide		1 (0.2%)
**Session with intra-DRG or other treatment**		85 (17.7%)

^a^ For inpatients, reimbursements of treatments with intra-DRG status are not available because the cost is included in the overall cost of the hospital stay

### Safety (*N* = 2,771)

In the cohort, 91.7% of patients (*n* = 2,542) were not hospitalized during the exposure period for one of the cardiovascular or bleeding events studied, and most others had only one hospitalization (*n* = 170, 6.1% of the CLL cohort). The proportion of patients hospitalized was higher among patients with a cardiovascular comorbidity recorded in the 12 months before ibrutinib initiation (5.9% versus 11.0%, p value < 0.0001) and those aged more than 70 years (5.9% versus 9.4%, p value < 0.0001).

The most frequent events were bleeding (*n* = 79, 2.9%), atrial fibrillation (*n* = 77, 2.8%) and heart failure (*n* = 74, 2.7%) (Table 4). For half of the patients who experienced at least one hospitalization, the first hospitalization occurred within 6 months of ibrutinib initiation (Q1-Q3: 2.4–2.9). More than half of these patients (*n* = 140, 61.1%) continued ibrutinib after their first hospitalization, with the first dispensation recorded within the 18 days of hospital discharge in median.

Over the 12 months following ibrutinib initiation, 18.1% (*n* = 502) of patients initiated a first dispensation of an antihypertensive (or received a second or third additional antihypertensive agent) or anticoagulant or antiplatelet drug (9.5%, 9.6% and 2.5%, respectively).

**Table 4:** Description of the onset of cardiovascular and bleeding events of interest during ibrutinib exposure

		CLL
		**(** ***N*** ** = 2,771)**
**Duration of ibrutinib exposure (months)**		
Mean (SD)		14.2 (11.4)
Median (Q1-Q3)		11.2 (4.2–22.5)
Min–Max		0.1–41.1
**Reason for end of ibrutinib exposure**		
End of study period		1,858 (67.1%)
Discontinuation		484 (17.5%)
Death		406 (14.7%)
Lost to follow-up		23 (0.8%)
**Patients with at least one hospitalization for a cardiovascular and bleeding event of interest**		229 (8.3%)
Bleeding		79 (2.9%)
Atrial fibrillation		77 (2.8%)
Heart failure		74 (2.7%)
Hypertension		11 (0.4%)
Cardiac arrest		6 (0.2%)
Paroxysmal tachycardia		4 (0.1%)
**Number of hospitalizations for a cardiovascular event of interest**		
0		2,542 (91.7%)
1		170 (6.1%)
2		41 (1.5%)
≥ 3		18 (0.6%)
**Time between the initiation of ibrutinib and the first hospitalization (in months)**		
N (%)		229 (8.3%)
Mean (SD)		8.9 (8.6)
Median (Q1 - Q3)		6.4 (2.4–12.9)
**Duration of the first hospitalization (in days)**		
Mean (SD)		7.6 (8.4)
Median (Q1 - Q3)		5.0 (2.0–10.0)
**Patients with at least one dispensation of ibrutinib after the first hospitalization** (***N* = 229)**		140 (61.1%)
**Time between the end of the first hospitalization and the following dispensation** **of ibrutinib (in days)**		
Mean (SD)		26.8 (29.3)
Median (Q1 - Q3)		18.0 (7.5–33.0)
**Patients initiating an antihypertensive, anticoagulant or antiplatelet or receiving a second or third additional antihypertensive agent in the 12 months following ibrutinib initiation**		502 (18.1%)
Initiation of antihypertensive or of a second or third additional antihypertensive agent		262 (9.5%)
Initiation of anticoagulant		265 (9.6%)
Initiation of antiplatelet		69 (2.5%)

### Effectiveness (*N* = 2,771)

**Fig. 2:**
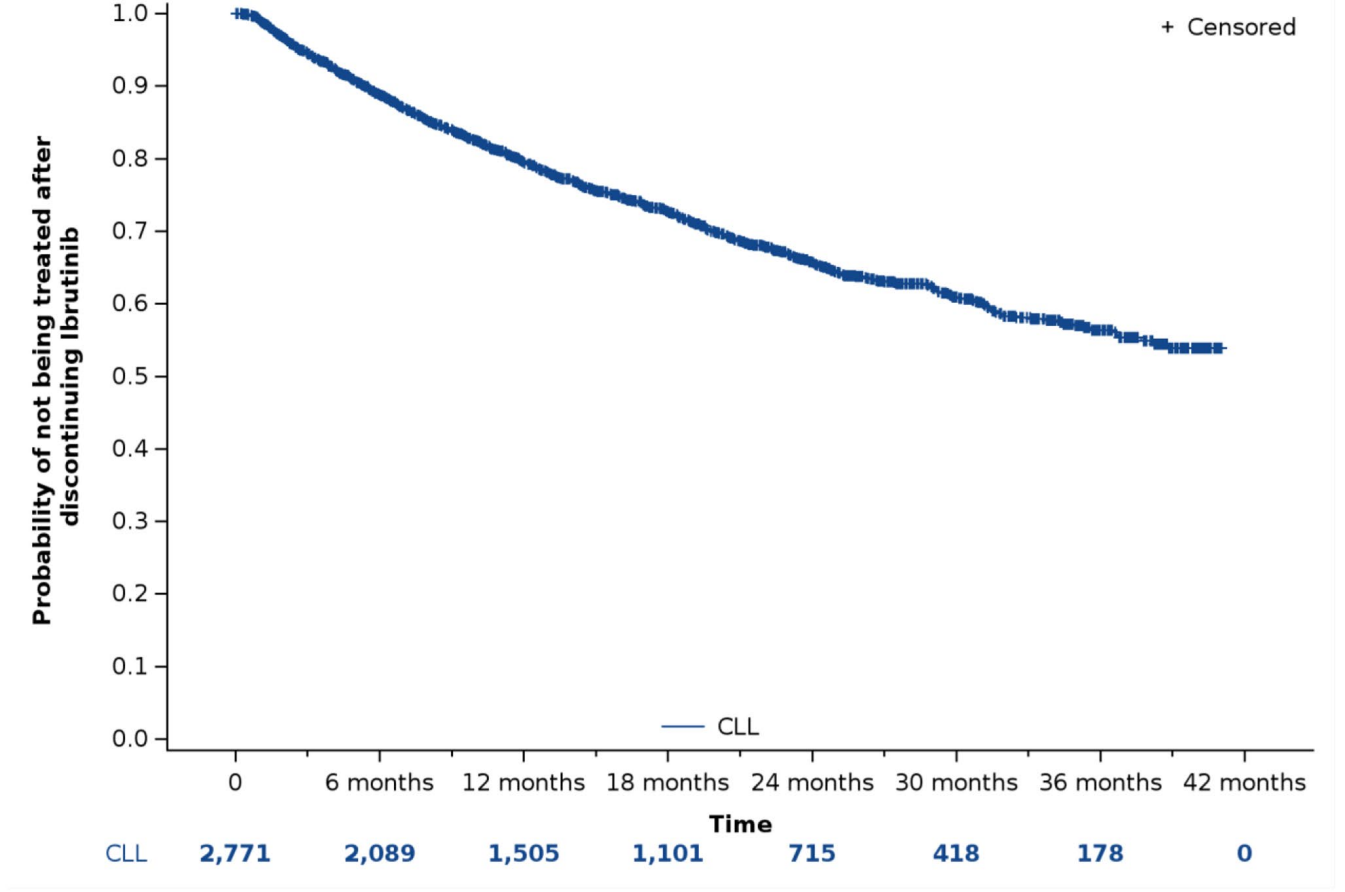
Kaplan‒Meier curve illustrating the time to next treatment in patients with CLL initiating ibrutinib (*N* = 2,771)

Among the cohort (*N* = 2,771), neither the median TTNT (Fig. 2) nor the median TTD was reached by the end of the study period. At 12, 24 and 36 months, the probability of a treatment change or death was 21%, 34% and 44%, respectively (with a percentage of deaths of 11%, 17% and 19%, respectively). At the same time points, the probability of permanent treatment discontinuation or death was 27%, 40% and 47%, respectively.

**Fig. 3:**
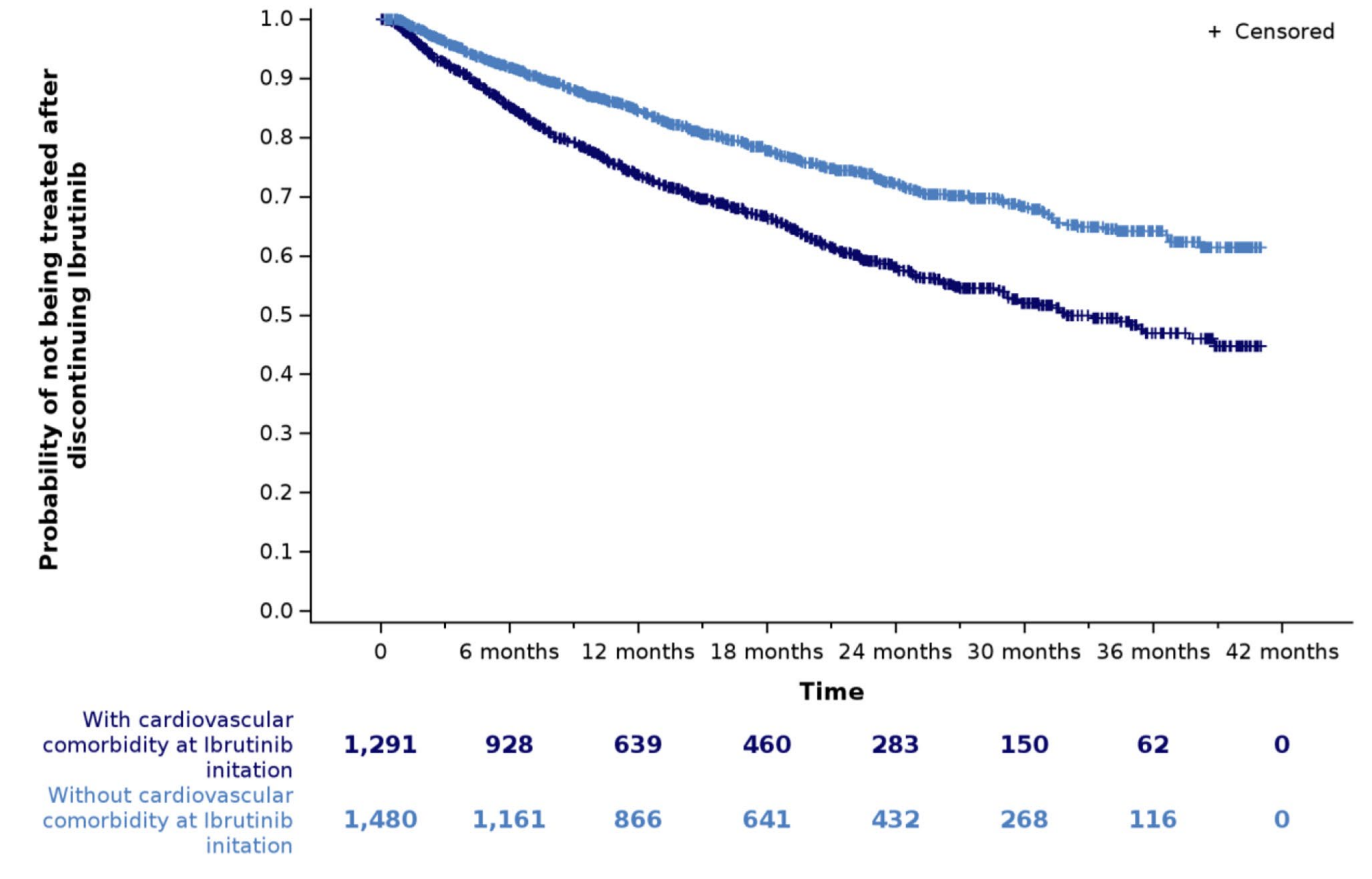
Kaplan‒Meier curve illustrating the time to next treatment in patients with CLL initiating ibrutinib, according to the presence of a cardiovascular comorbidity at ibrutinib initiation

The median TTNT was 32 months for patients with a cardiovascular comorbidity at the index date (*N* = 1,291) and was not reached for patients without (*N* = 1,480) (Fig. 3). In patients with a cardiovascular comorbidity, the probability of a treatment change or death at 12, 24 and 36 months was 26%, 42% and 53%, respectively (with a percentage of deaths of 15%, 23% and 26%, respectively). Among patients without a cardiovascular comorbidity, the probability of a treatment change or death at 12, 24 and 36 months was 16%, 28% and 36%, respectively (with a percentage of deaths of 7%, 11% and 13%, respectively).

When studying explanatory factors of the TTNT, a higher risk was observed for patients aged 70 years and more versus less than 65 years (HR = 1.36, 95% CI: [1.10–1.67]), for patients with a cardiovascular comorbidity at ibrutinib initiation (HR = 1.49, 95% CI: [1.28–1.72]), for patients with a hospitalization for a cardiovascular or bleeding event studied during ibrutinib exposure (HR = 3.04, 95% CI: [2.43–3.79]) and for those initiating ibrutinib as L2+ (HR = 1.39, 95% CI: [1.13–1.71]) (Table 5). Conversely, a decreased risk for patients who initiated an antihypertensive or anticoagulant drug or had a second or third antihypertensive agent added in the 12 months following ibrutinib initiation (HR: 0.66, 95% CI [0.54–0.81]) was noted.

**Table 5:** Explanatory factors of the time to the next treatment using a Cox proportional hazard model (*N* = 2,771)

	Unadjusted model				Adjusted model		
	HR	95% CI	*p* value		HR	95% CI	*p* value
**Sex**							
Male	1.00	-	-	.	1.00	-	-
Female	0.82	[0.71–0.96]	0.0112	.	0.88	[0.75–1.02]	0.0849
**Age at index date**							
< 65	1.00	-	-	.	1.00	-	-
65–69	1.05	[0.81–1.38]	0.6967	.	1.01	[0.77–1.32]	0.9673
≥ 70	1.52	[1.24–1.86]	< 0.0001	.	1.36	[1.10–1.67]	0.0037
**Cardiovascular comorbidity at inclusion**							
Absence	1.00	-	-	.	1.00	-	-
Presence	1.70	[1.48–1.96]	< 0.0001	.	1.49	[1.28–1.72]	< 0.0001
**Hospitalizations for cardiovascular or bleeding event studied during ibrutinib exposure** ^**a**^							
Absence	1.00	-	-	.	1.00	-	-
Presence	3.02	[2.44–3.73]	< 0.0001		3.04	[2.43–3.79]	< 0.0001
**Line of treatment (L1/L2+)**							
L1	1.00	-	-		1.00	-	-
L2+	1.33	[1.08–1.64]	0.0066		1.39	[1.13–1.71]	0.0020
**Initiation of an antihypertensive or anticoagulant or addition of a second or third** **antihypertensive agent** ^**b**^							
Absence	1.00	-	-	.	1.00	-	-
Presence	0.79	[0.65–0.96]	0.0173	.	0.66	[0.54–0.81]	< 0.0001

^a^ Time-dependent variable

^b^The hypothesis of proportionality of risks is not satisfied, the observed effect is an average effect over the period of the study

## Discussion

### Main findings

The profile of ibrutinib users was in accordance with the marketing authorization and reimbursement during the analysed period. A predominant pattern of use was identified: ibrutinib was mostly used in L2+ (with increased L1 use over the years) and as a monotherapy, which is consistent with the reimbursement label during that time frame. This study also confirmed effectiveness and safety data. The median TTNT was not reached, and the probability of a treatment change or death was 21%, 34% and 44% at 12, 24 and 36 months, respectively. Only 8.3% of patients were hospitalized for a cardiovascular or bleeding event, of whom more than half continued to be treated with ibrutinib after hospitalization.

### Internal and external validity

Patients included in this study can have high-risk profile (TP53/del17p mutation and/or relapse/refractory CLL). Patient characteristics (more males and elderly) corresponded to those of patients with CLL [21] and of ibrutinib users in other real-life studies [22–24]. A high comorbidity burden at ibrutinib initiation was observed, as would be expected in an elderly population. Indeed, even if ibrutinib is associated with some cardiovascular events [14–16], almost half (46.6%) of patients had a diagnosis or another probabilistic marker (anticoagulant, antiplatelet or pacemaker) of cardiovascular disease before ibrutinib initiation. One-fifth of patients had a solid tumour in addition to leukaemia. The three most frequent corresponded to the two most frequent types of cancer in the French population (breast and prostate) [25] and to skin cancer (i.e., melanoma and carcinoma).

Considering that this elderly population has a high cardiovascular burden, the percentage of patients hospitalized for a cardiovascular event was low (8.3%). However, this analysis was performed over the exposure period, whether treatment ended or not. Consequently, patients who were still exposed to ibrutinib at the end of the study period and who were hospitalized thereafter were not considered. Most patients continued ibrutinib after being hospitalized for a cardiovascular event, suggesting that in most cases, the cardiovascular event was probably not severe or not related to ibrutinib. As expected, patients were seldom hospitalized for hypertension (0.4%), but 9% started antihypertensive treatment or experienced an increase in hypertensive therapy over the 12 months following ibrutinib initiation. This is consistent with the frequency of hypertension onset described in the clinical trial RESONATE 2 (12% of patients experienced hypertension of any grade during the first year of ibrutinib treatment) [26, 27]. On the other hand, two retrospective noninterventional studies suggested a high burden of hypertension, for example, Binsah et al. observed that nearly 23% of ibrutinib users developed new or worsened hypertension [28, 29]. Unlike this study, hypertension was identified by blood pressure measures, which may explain why the percentages were higher than those in this study. The proportion of patients with bleeding onset in this study (2.9%) was lower than that in the RESONATE 2 study (11% over a median treatment duration of 57 months, including 4% and 3% in years 0–1 and 0–2, respectively). Moreover, patients with CLL are at an increased risk for major haemorrhage [30] because of the disease itself, comorbid conditions, concomitant medications or clinically significant thrombocytopenia [31]. Indeed, this study showed that bleeding events were more frequent in patients with a cardiovascular comorbidity at ibrutinib initiation (p value < 0.0001). In the SNDS database, the cause of haemorrhage, and thus the potential causal link between haemorrhage and ibrutinib, is not available, preventing us from hypothesising further. Conversely, the occurrence of atrial fibrillation was not significantly different between patients with and without a cardiovascular comorbidity, as described in clinical trials and real-world studies [22, 32]. Atrial fibrillation was also less frequent in this study (2.8%) than in the RESONATE 2 study (6% in years 0–1 and 1% in years 1–2), which may be explained by atrial fibrillations being identified from hospitalisations (diagnoses from cardiologist visits are not available). However, it is noteworthy that 9.5% of patients initiated anticoagulant therapy within 12 months of ibrutinib initiation, some cases of which may be related to the management of atrial fibrillation. Indeed, the treatment and prevention of atrial fibrillation in patients taking ibrutinib often requires anticoagulation therapy [33].

Effectiveness was assessed through the TTNT, which was used as a proxy for PFS. The median PFS was 44.1 months in the RESONATE study, which included patients with previously treated CLL/small lymphocytic lymphoma, and was not reached in the RESONATE 2 study, which included previously untreated patients. Consistent with the lower median follow-up in this study (16.2 months vs. 65.3 and 60 in RESONATE and RESONATE 2, respectively), the median TTNT was not reached and the median duration of ibrutinib therapy was low (6.0 months versus 41 months in the RESONATE study). Indeed, only patients who discontinued ibrutinib early were able to be identified and patients with a long duration of treatment were still being treated at the end of their follow-up. However, the median TTNT was reached for patients with a cardiovascular comorbidity at ibrutinib initiation (median TTNT of 32 months), for whom this study revealed a higher risk of a treatment change or death (HR: 1.49, 95% CI [1.28–1.72]). This risk was also higher for patients who were hospitalized for a cardiovascular or bleeding event (HR: 3.04, 95% CI [2.43–3.79]), as well as for patients older than 70 and patients initiating ibrutinib as L2+. Conversely, patients who initiated an antihypertensive or anticoagulant or who received an additional second or third antihypertensive agent had a lower risk of a treatment change or death (HR: 0.66, 95% CI [0.54–0.81]). Dickerson et al. showed that the initiation of an antihypertensive medication after hypertension development was associated with a lower risk of subsequent major adverse cardiovascular events, which could be a hypothesis explaining this lower risk of a treatment change or death [29]. This protective effect against a treatment change or death may be a first reassuring element in the use of anticoagulants in ibrutinib patients, whose prescription may be associated with a clinical challenge in balancing the risk of thrombosis and bleeding [33] and would benefit from further investigations.

### Limitations

Some limitations can be highlighted. Indeed, the indication for which ibrutinib was prescribed is not recorded in the SNDS. Therefore, an algorithm was developed to identify it, which was designed to be restrictive on certain definitions to avoid any overinterpretation. This algorithm has not been validated. The first limitation is related to some unspecific haematologic cancer codes that can potentially be used for coding CLL. Patients for whom only these codes were recorded were not included in this study. Second, chemotherapy reimbursements with an intra-DRG status are not available in the SNDS database, which makes it impossible to know precisely which chemotherapy protocols the patient has received before, concomitantly with and after ibrutinib. The unavailability of results of lab tests in the SNDS also makes it impossible to determine the patient’s mutational status (i.e., the presence of a 17p deletion or TP53 mutation) and therefore the exact indication for which ibrutinib was prescribed. Last, the SNDS does not allow the cause of death or whether treatment was related to death to be determined.

Other limitations impacted the description of the pattern of ibrutinib use. Indeed, the dosage is also not available in the SNDS database, which constituted an issue in determining the period of ibrutinib coverage and in differentiating a decreased posology from a standard posology with a period of discontinuation. Consequently, the gap was based on the lower posology used in practice (1 capsule per day) to not consider a decrease in posology as a discontinuation. However, with this definition, short periods of discontinuation cannot be identified. Additionally, there are no data about the progression of disease in the SNDS allowing the estimation of PFS, which was therefore approximated by estimation of the TTNT. Last, the description of the treatment history and concomitant and next treatments were available only for patients who completed their first regimen. Thus, this analysis is not representative of the overall population of ibrutinib users if the use of these treatments is different between patients with a short regimen and those with a long regimen that had not been completed at the time of the analysis. Last, treatment-related adverse events cannot be identified in the SNDS database, as well as the indication for which treatment has been prescribed. Hence, an association between ibrutinib and the occurrence of a cardiovascular event or cardiovascular treatment cannot be established.

### Strengths

The SNDS database is large and representative of the French population. This is a comprehensive claims database, covering 98.8% of the French population and registering all dispensed and reimbursed drug prescriptions. Even if we focused only on patients covered by the general health insurance scheme or local mutualist section (approximately 80% of the French population), this database has led to the constitution of the largest cohort of CLL patients using ibrutinib in the world. Thanks to hospital diagnoses, LTD status, treatments and medical devices, the SNDS database has provided us with robust data on the patient’s general health status, especially their comorbidities. They also enabled us to calculate the Charlson score accurately, using the ICD-10 comorbidity coding algorithm developed and validated by Quan et al. in administrative data [34].

## Conclusion

This study constituted one of the largest cohorts of ibrutinib patients in the world, including 2,771 CLL patients. The size and representativeness of the dataset facilitates reliable trends of results. The profile of ibrutinib users was in accordance with the marketing authorization and reimbursement during the analysed period. This study also confirmed effectiveness and safety data.

### Electronic supplementary material

Below is the link to the electronic supplementary material.

Supplementary Material 1

## Data Availability

The datasets presented in this article are not readily available because, due to NHS and SNDS rules, no data sharing is possible as access to data is restricted to habilitated and qualified researchers (Floriane Deygas is habilitated and qualified).
