# Supplementary material for: A retrospective observational study of ibrutinib in chronic lymphocytic leukaemia in a real-life setting in France using the national claims database (OSIRIS)
Source: Ann Hematol. 2024 Jul 5;103(8):2969–81. doi: 10.1007/s00277-024-05859-w (PMC11283383; doi:10.1007/s00277-024-05859-w)
Supplement: Supplementary file 1 — Supplementary Material 1 [file 277_2024_5859_MOESM1_ESM.docx]

**Supplementary Information**

**Annals of hematology**

**A retrospective observational study of ibrutinib in chronic lymphocytic leukaemia in a real-life setting in France using the French claims database (OSIRIS)**

Sylvain Choquet^1^, Clarisse Marchal^2^, Floriane Deygas^2^, Marine Deslandes^3^, Nahid Macher^3^, Gérard de Pouvourville^4^, Vincent Levy^5^

Corresponding author:

Clarisse Marchal, PELYON, Lyon, France;

Email: clarisse.marchal@pelyon.fr

**Online Resource 1: Algorithm defining the line of treatment and the ibrutinib regimen**

The use of ibrutinib was considered as first line (L1) if there was no dispensation of a CLL-specific treatment (listed in Supplementary Table A3) or infusion session at a hospital between January 1^st^, 2006, and 14 days before the index date; otherwise, the use was considered as second or more line (L2+). The ibrutinib coverage period was defined as the ratio of the number of capsules dispensed to the ibrutinib-specific dosage for CLL (i.e., 3 capsules per day). A gap of more than 90 days (when dispensing a 90-capsule package) or 110 days (when dispensing a 120-capsule package) without an ibrutinib dispensation after the end of the coverage period was considered to indicate discontinuation. Discontinuation was considered permanent when the ibrutinib discontinuation period was more than 6 months and temporary otherwise. An ibrutinib regimen was defined as the period from the date of the first ibrutinib dispensation (index date) to the identification of discontinuation (permanent or temporary) or the patient’s death, whichever came first. Any CLL-specific treatment dispensed during or within 14 days prior to the start of the ibrutinib regimen was considered a concomitant therapy.

**Online Resource 2: CLL-specific treatments**

| **Treatment class** | **Product** |
| --- | --- |
| **Chemotherapy** | Cyclophosphamide |
|  | Chlorambucil |
|  | Fludarabine |
|  | Vincristine |
|  | Bendamustine |
|  | Cisplatin |
|  | Doxorubicin |
| **Targeted therapy** | Idelalisib |
|  | Venetoclax |
|  | Acalabrutinib |
| **Immunotherapy** | Rituximab |
|  | Obinutuzumab |
|  | Ofatumumab |
|  | Alemtuzumab |
